# A System-Level Approach towards a Hybrid Energy Harvesting Glove

**DOI:** 10.3390/s21165349

**Published:** 2021-08-08

**Authors:** Emad Iranmanesh, Weiwei Li, Hang Zhou, Kai Wang

**Affiliations:** 1 School of Electronic and Computer Engineering, Peking University Shenzhen Graduate School, Shenzhen 518055, China; iranmanesh.emad@yahoo.com (E.I.); zhouh81@pku.edu.cn (H.Z.); 2 Key Laboratory of Microelectronics Device and Integrated Technology, Institute of Microelectronics, Chinese Academy of Sciences, Beijing 100029, China; liweiwei2020@ime.ac.cn; 3 School of Electronics and Information Technology, Sun Yat-Sen University, Guangzhou 510006, China

**Keywords:** hybrid harvesting system, wearable electronics, piezoelectric charge-gated thin film transistor, photoelectric effect

## Abstract

This paper presents a novel wearable hybrid harvester system as a glove that contains four distinct scavenging modules of flexible transducer film, photosensitive 3D dual-gate thin-film transistor, and a particular power management box. Each single module is formed by a piezoelectric-charge-gated TFT (PCGTFT). The reported system is capable of scavenging energy from two various free of charge energy sources (Piezoelectric plus Photoelectric). Aforesaid system unlike other state-of-the-arts overcomes several key challenges in interfacing, storage and power management. Harvested energy which is administered through power and storage management system ultimately lightens a typical light emitting diode (LED), testifies capability of such glove to power up some low-power electronic devices.

## 1. Introduction

Wearable electronic devices such as newly introduced gadgets including smart watches, bands or glasses brought by Google, Apple and some other companies, are widely received lots of attention recently. Scavenging energy through wearables plays a vital role in powering up suchlike devices rather than utilizing conventional, bulky, and unsafe lithium batteries. Although various harvesters in general terms target to scavenge energy from different sources [1,2], the most common strategy in wearables is to focus on non-gratis body vibrations. Piezoelectric-based scavengers are well adopted units in vibration-based wearable systems [3,4]. Aforementioned systems technically consist of several components classifying into harvesting unit, voltage regulating circuit, and power management board [5,6]. Generally, gentle and repeatable body movements practically are referred to low-frequency vibrations from mechanical stand point. Such single-phase harvesting systems yet suffer from low power density. This issue specifically in wearables is well-prevailed through focusing on hybrid harvesters which scavenge energy from two or several sources. Employing a piezo-harvester as the harvesting unit in this case, results in scavenging the energy sourcing from low-frequency human motions. The obtained AC signal needs to get regulated into DC utilizing a rectification circuit. This could be very problematic where a harvesting system with couple of scavenging modules requires specific regulating circuits allocated to each [7,8,9,10,11]. As the signal is regulated, it needs to be stored somehow through a power management box and interfacing between the harvesting unit and the power management board may need an innovative design and structure. Implementation of such wearable system yet suffers from several challenging key points. Reports in this case pointed out some sophisticated issues as: simultaneous harvesting energy from two or several sources, Interfacing between harvesting unit and the management board, complexity of rectification circuit, and preventing any unspecified damages to management board due to any mechanical shock originating from human body movements [12,13,14,15]. Recent progress to overcome aforementioned issues may also become hectic since having an interface unit along with out-system rectification circuits makes the wearable system complexed and spacy [16,17,18]. Moreover, since the output of harvesting units may differ based on the application and type of harvesting, interconnection between several scavenging units is considered problematic. We previously reported a unique single-mode wearable harvesting module based on a piezoelectric charge generator biased a dual-gate TFT [19]. The functioning principle of reported module shows that initially mechanical stimuli in terms of body motions results in charge generation in piezoelectric material (PolyVinylidene Fluoride-PVDF). Obtained AC signal in the following inputs the DGTFT which works well in its saturation region. Top gate, bottom gate and drain are shorted and biased via bottom electrode of PVDF transducer film where source is considered as output channel and threshold voltage is given as 2 V. DGTFT not only acts as a rectifier to regulate the AC signal, but also functions well as a buffer to prevent any damage may occur by a mechanical shock to IC peripheral. Integration of such harvesting module has been implemented while the challenging key points have also been addressed [20]. In the next step, given harvesting module has also been re-designed to form a hybrid harvester which scavenges energy from two distinct energy sources simultaneously (both from dynamic body vibrations and light exposure). Evolution of aforesaid harvesting module to form a hybrid scavenger has been fulfilled by replacing a 3D photosensitive dual-gate TFT as a rectifier [20].

**Figure 1:**
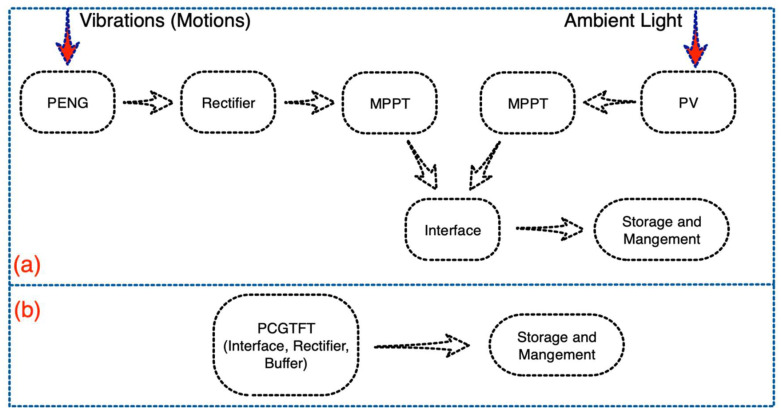
(**a**) Components of hybrid harvester (piezoelectric and photovoltaic); (**b**) System layout of proposed hybrid harvesting system.

Taking solar and motions as two distinct energy sources for wearable hybrid harvesters, implies priority of proposed system compared to its counterparts. Figure 1a illustrates how a typical scavenger containing of two main elements as photovoltaic and piezoelectric functions in a system level approach. As mentioned earlier, DC output of photovoltaic is needless of any rectification while the AC signal from piezoelectric needs to be regulated. Moreover, each energy harvester includes a MPPT circuit, which dynamically adjusts the operational parameters of the energy conversion devices in response to the variations of energy sources so that the output power can be maximized. Some articles recently addressed a novel method in power management and storage system regarding to hybrid harvesters, but the mismatching between different scavenging modes and interfacing among them are yet required improvements [21].

Looking at Figure 1b verifies ease of interfacing and power management circuit design which could be quite interesting in terms of wearables. Unlike other widely used hybrid (multi-mode) harvesters reported recently [22,23], proposal of a hybrid wearable scavenger system consists of several dual-mode harvesting modules sounds promising to scavenge energy from two distinct sources simultaneously. Since the DGTFTs in each harvesting module as the backbone of harvester, function as rectifier, the appointed state-of-the-art harvesting system is needless of any extra sophisticated regulating circuits and design. In addition, buffer-like TFT unlike a Schottky diode prevents voltage breakdown which may affect later the management board and peripheral IC circuits. Employing a diode-like TFT in the harvesting system also elevates the problem of interfacing between the harvesting module and the power management board. Therefore, the whole system is needless of any extra components which results in having a compact system with no complexity in circuits. Recent published data testified that internal resistance of DGTFT plays a key role in device characterization, particularly as a photosensitive device. The aforementioned DGTFT internal resistance is negligible compared to utilized load resistor on which the signal is monitored across. 

Table 1 contrasts several main features of singular-phase and hybrid harvesting systems from various sources of energy. It describes well, why widely-used hybrid scavenging systems yet require improvement. The current paper reports on a system-level approach towards a novel hybrid wearable harvesting glove. It details a multi-mode harvesting glove consisting of four PCGTFTs along with designing a specific power management board which is compatible with the particular system. It not only demonstrates principles of harvesting through two different energy sources simultaneously, but also presents how effectively the efficiency and system internal resistance are associated. Finally, the obtained output power of the system in particular time-domain of utilizing well-approves the concept of powering up some low-power electronic devices, though the management system yet needs evolution.

## 2. Methods

### 2.1. Device Architecture

**Figure 2:**
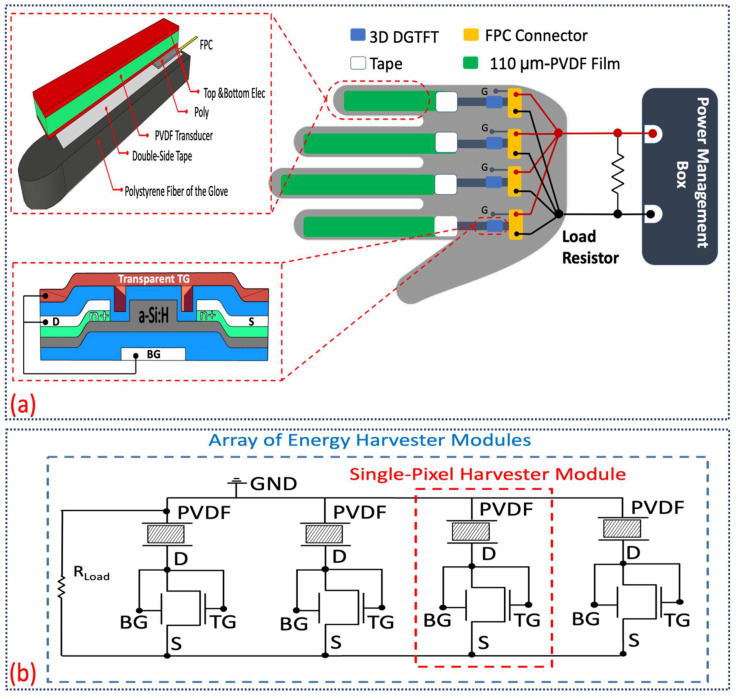
(**a**) Schematic diagram of hybrid glove along with its primary components. Dual-mode harvesting formed by a diode-connected 3-D photosensitive DGTFT biased polyvinylidene difluoride (PVDF) transducer; (**b**) equivalent circuit of harvesting system containing of four modules.

A clear sketch on how the hybrid glove is formed along with the schematic layout of four harvesting modules are depicted in Figure 2a. Major components of the proposed wearable harvesting system are classified into the harvesting modules which shape a glove, interfacing between the glove and the power management box, and power management board itself. A 3D photosensitive dual-gate TFT is also illustrated in Figure 2a. It fairly suggests simply how to attach each harvesting module (PCGTFT) into the polystyrene fiber-based glove. The equivalent electrical circuit of aforesaid system in form of an array has been explained in Figure 2b. The concept of having dual-mode harvesting modules basically is firmed on introduction of photosensitive 3D DGTFT [31]. Characterization and impact of ambient light on such dual-mode units have already been presented. In the current paper, shaping a hybrid harvesting glove is targeted thus, fabrication of 3D diode-like TFT, lamination of transducer film and integration of each harvesting module will be focused initially. Thereafter, the design structure of scavenging system in form of a glove with respect to interfacing, power management and IC-circuits will be addressed.

As shown above, Figure 2a describes that DGTFT is integrated with 110 μm-PVDF film to from a PCGTFT. Due to any mechanical stimuli, generated charges through PVDF transducer flow, and consequently bias the DGTFT with several tens of volts. Threshold voltage of fabricated 3D DGTFT ranges between 1–3 V. Top gate, bottom gate, and drain are shorted where source acts as the output. In this scenario, TFT not only functions well in saturation region to rectify the AC signal but also, works as a buffer to prevent any unexpected damage on IC peripheral circuits. These features result in overcoming rectification/buffering circuit complexity, and common interfacing issues. The output power and efficiency of piezo-based harvesting module will be enhanced due to decrement in DGTFT internal resistance when an ITO transparent electrode atop each DGTFT allows photon absorption. In this case, device scavenges energy not solely from mechanical stimuli but also, from ambient light. Moreover, the schemed device unlike other reported multi-mode wearable harvesters, scavenges energy simultaneously needless of any extra unit for management or synchronization. Raised key points about dual-mode harvesting module effectively dominate system intricacy and spacey once the hybrid glove is taken into account. Therefore, proposal of forming a hybrid glove through several dual-mode harvesting modules sounds promising to enhance the system performance and prevail general unsolved key issues in widely reported multiple-mode harvesting systems. As expected, internal resistance of DGTFT follows the same reduction trend in a system level approach. This decrement leads to greater obtained voltage and ultimately enhances the system efficiency. Aforementioned hybrid glove has been formed and tested experimentally where load is set as 10 MΩ. Output power is stored well in a super-capacitor mounted on management board. The extracted data which will be discussed later, testify the capability of such system to power up some low-power electronic devices like commercially-available LEDs.

### 2.2. Integrated Harvesting System

#### 2.2.1. Transducer Lamination

**Figure 3:**
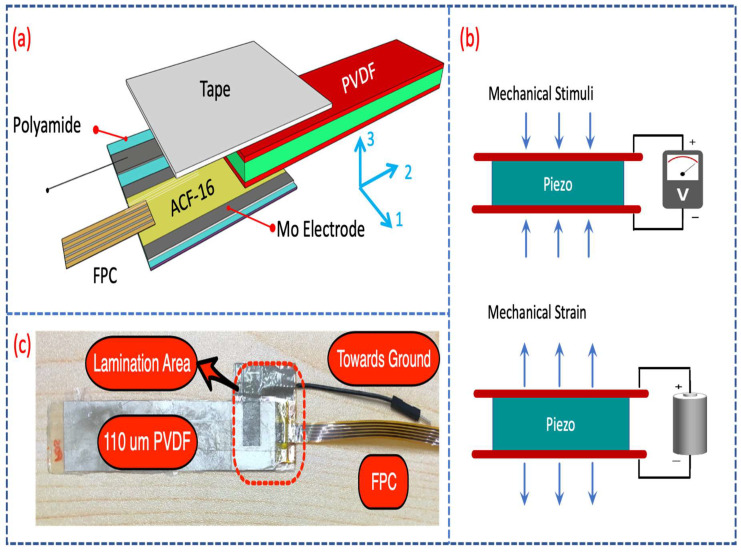
(**a**) Schematic lamination of thin-film flexible transducer part on a polyamide substrate. The given coordinate system in 1, 2 and 3 directions is used to analyze the numerical modeling of charge careers in piezoelectric-material; (**b**) Principles of piezoelectricity in schematics; (**c**) Laminated 110 μm-PVDF.

Necessity of several integrated dual-mode harvesting modules to form a hybrid scavenging system entails remarkable probe on lamination of transducer part as the initial step. Figure 3a depicts the schematic structure of a single transducer film which is laminated on a flexible substrate. An Ag/110-μm PVDF/Ag sandwich structure film is used to laminate on a polyamide substrate on which Molybdenum (Mo) electrodes have already been coated. Conductivity of electrodes are measured and reported in range of 1–5 ohm. Generally, lamination process is predicated where two distinct edges, surfaces, or planes are involved to bond through applying a certain adhesive. If conductivity matters, the process sorts out by utilizing a conductive adhesive. Aforesaid procedure practically requires two significant technical factors called as pressure and temperature [32]. Effective pressure in this scenario ranges between several tens of Pascals to several MPas while the bonding temperature is rationally tuned as 120 °C to 250 °C based on various applications. In case of proposed structure containing a PVDF film as transducer, high temperature lamination causes polarization loss in piezoelectric materials and consequently damages the PVDF transducer. Therefore, adopting a low-temperature lamination process compatible with PVDF film with sound pressure exertion is of a great necessity. Temperature-characterization of PVDF transducer dictates 70 °C as the setting point not to lose polarization. Finally, meeting an isotropic conductive film (ACF-16) identical to low-temperature lamination is desired. Pre-lamination of ACF-16 for acceptable bonding is done under 70 °C and 0.1 MPa for several seconds. This initial step cooperates to crash the conductive nano-particles and builds a proper bonding. As shown in Figure 3a, ACF will be laid on a 1 cm × 1 cm-cotaed electrode on polyamide and 110-μm PVDF film is well-aligned atop. An FPC connector also is placed and firmly located atop the ACF utilizing a commercially-available tape. Laminator machine, TWB-150; KeFu Instrument, is used to implement lamination process (Supplementary File S1). The lowest lamination temperature of ACF-16 for effective bonding limits to almost 100 °C. Therefore, compensating setting temperature at 70 °C by tuning the pressure to 0.3 Mpa is evaluated. Applied pressure on the target area covered by ACF-16 results in crashing nano-particles and grounds a proper conductive bonding. Hence, having a corridor to guide the charge careers flowing from PVDF film due to any expansion or compression is implemented. Charge generation of PVDF requires an analytical modeling to study how DGTFT is biased through PVDF. 

Regarding to principles of piezoelectricity illustrated in Figure 3b, (conversely to charge generation due to exertion of pressure on piezo-materials, mechanical strain occurs as a result of any applied biases), applied dynamic stimuli on the laminated transducer thin-film results in charge generation. The negative/positive charge accumulation near to top/bottom electrodes of PVDF due to polarization in 31 and 33 directions (illustrated in Figure 3a) can be finally quantified as:

(1) $$d_{33}=\frac{electrical\;charge\;density}{applied\;stress}=\frac{Q_{33}/A_{3}}{F_{33}/A_{3}}$$

where *Q*_33_ is the generated charge, *A*_3_ is area of the surface that is perpendicular to the force, and *F*_33_ is the applied force in 33 direction. Therefore,

(2) $$Q_{33}=d_{33}\cdot F_{33},$$

Considering the piezoelectric-material capacitance results in:

(3) $$C_{33}=\frac{\varepsilon \cdot A_{33}}{t}=\frac{\varepsilon \cdot W\cdot L}{t},$$

where *ε* is permittivity, *t* is thickness, *L* is the length, and *W* is the width of the piezo-film respectively. The open circuit voltage can be given as:

(4) $$V_{33}=\frac{Q_{33}}{C_{33}}=d_{33}\cdot F_{33}\cdot \frac{t}{\varepsilon \cdot W\cdot L},$$

The same procedure may follow for generated charge in 31 direction.

(5) $$d_{31}=\frac{electrical\;charge\;density}{applied\;stress}=\frac{Q_{31}/A_{3}}{F_{31}/A_{1}}=\frac{Q_{31}\cdot t}{F_{31}\cdot W}$$

where *Q*_31_ is the generated charge, *A*_1_ is area of the surface that is perpendicular to the force, *t* is the thickness, *W* is the width of PVDF film, and *F*_31_ is the applied force in 31 direction.

(6) $$Q_{31}=\frac{d_{31}\cdot W}{F_{31}\cdot t},$$

The capacitor of piezoelectric-material in this direction is given as;

(7) $$C_{31}=\frac{\varepsilon \cdot A_{31}}{t}=\frac{\varepsilon \cdot W\cdot L}{t},$$

(8) $$V_{31}=\frac{Q_{31}}{C_{31}}=\frac{d_{31}\cdot W}{t\cdot F_{31}}\cdot \frac{t}{\varepsilon \cdot W\cdot L},$$

As it can be seen the material properties of piezoelectric plays a key role for charge generation. Therefore, to review the properties of utilized PVDF transducer and comment on its thickness selection in case of the proposed harvesting module, Supplementary File S2 is given.

#### 2.2.2. Fabrication of Photosensitive 3D Dual-Gate Thin-Film Transistor

**Figure 4:**
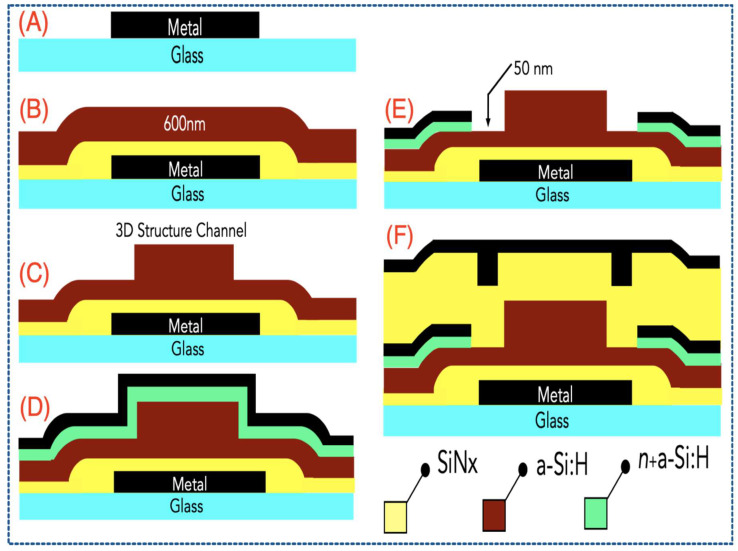
Fabrication steps of dual-gate TFT consisting of six vital processes labeling from (**A**–**F**).

It is shown previously that diode-like connected 3D DGTFT is an essential regulator and buffer for each harvesting module. 3D photosensitive dual-gate TFT enables harvesting from another mode apart from the mechanical stimuli. In this section, fabrication of such TFT on a glass substrate along with its characterization will be discussed. The dual-gate TFT was fabricated on the glass substrate by a series of processes which are well in line with commercial/industrial mainstream of TFT-LCD manufacturing. Figure 4 briefly illustrates required chronological steps. These 6 steps can be shortly classified. First, the bottom electrode was formed by deposition and patterning of Cr on a glass substrate. Then a 300-nm SiN_x_ dielectric layer and a 600-nm a-Si:H layer were both deposited consecutively. Dry etching the active layer and patterning the 3D FIN shape are then called. Part D and E in Figure 4 show that a 50-nm n^+^ a-Si:H and 100-nm Mo are deposited. Source and drain electrodes are created by wet etching and n+ layer is patterned by dry etching to form ohmic contact. Finally, top SiN_x_ dielectric layer and top gate transparent electrode (ITO) are deposited (F).

**Figure 5:**
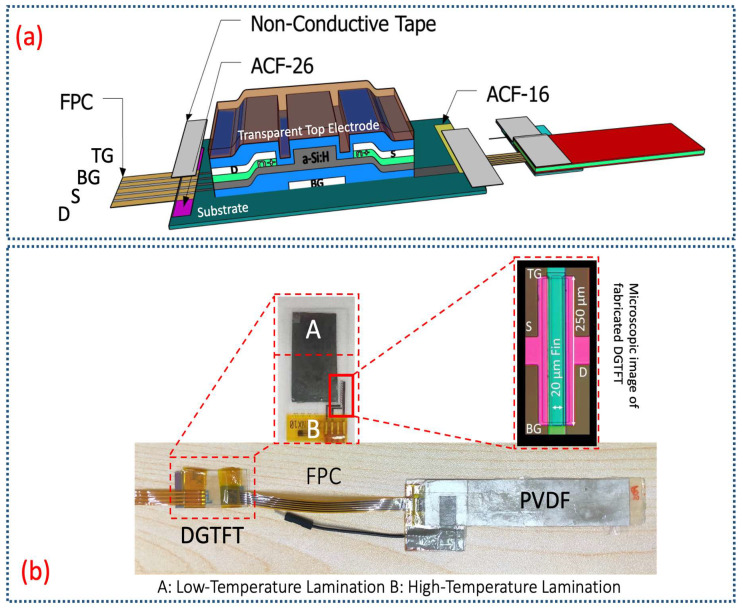
(**a**) Schematic of PCGTFT; (**b**) Experimental PCGTFT forming a single-pixel dual-mode harvesting module by integration of fabricated DGTFT and the laminated thin-film transducer on polyamide substrate.

Prior to characterization of 3D DGTFT, a single harvesting module has been integrated utilizing previously laminated transducer along with the fabricated TFT. Figure 5a depicts schematic of integration process while Figure 5b refers to experimental integrated module. Two different types of ACF (low/high-temperature process) were employed for proper bonding. The high temperature bonding has been implemented under 160 °C and the same pressure as discussed in low-temperature lamination. The electrodes on both sides of substrate on which the DGTFT is fabricated are coated utilizing Mo. To firmly attach the FPC connectors a sort of non-conductive tape is used atop.

TFT functions in saturation region once the applied stress through any dynamic motion like flexion turns it on. In a single harvesting module *TG*, *BG*, and Drain are shorted. Therefore, *V_TG_* = *V_BG_* = *V_DS_*. Taking Equations (2), (4), (6) and (8) results in:

(9) $$V_{TG}=V_{BG}=V_{DS}=\frac{d_{33}\cdot F_{33}+(\frac{l}{t})d_{31}\cdot F_{31}}{C_{Top}+C_{Bottom}+C_{Channel}},$$

where $C_{TOP}$, $C_{Bottom}$ and $C_{Channel}$ are termed as the top gate capacitance, the bottom gate capacitance, and the channel capacitance, respectively. Applied flexion on transducer in 33 and 31 directions are given as $F_{33}$ and $F_{31}$. $d_{33}$ and $d_{31}$ are the piezoelectric coefficients in 33 and 31 directions. Length (*l*) and thickness (*t*) of the transducer film are shown in Figure 3a. Modeling of such a single-pixel dual-mode harvester and the internal resistance of fabricated device are theoretically reported [33].

**Figure 6:**
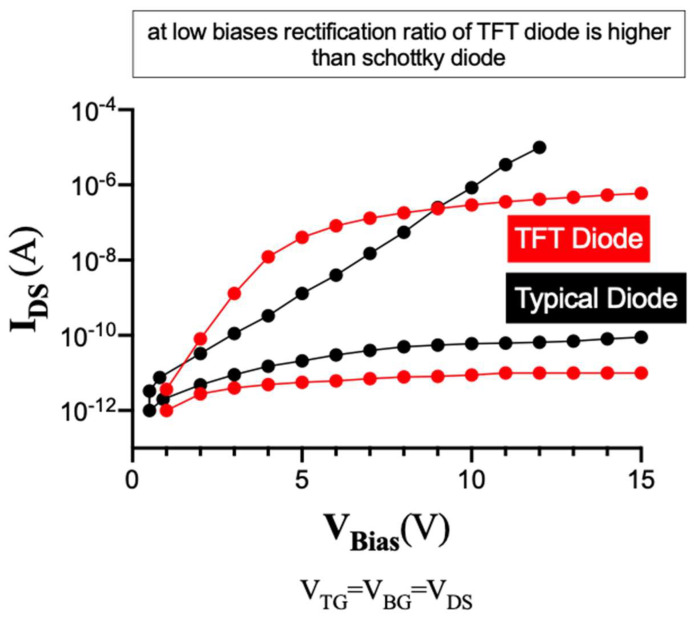
Rectification ratio analysis between diode-like TFT and a typical Schottky diode.

Employed 3D DGTFT differs from a Schottky diode in that diode-like TFT has higher rectification ratio than Schottky diode. The on/off ratio of the TFT can reach as high as 10^7^, more than 3–4 orders magnitude higher than that of the Schottky diode. The Schottky diode-based rectifier also suffers from high reverse current. Moreover, if a mechanical shock generates a high voltage, breakdown may occur which consequently damages the IC circuits. The diode-like TFT can generally tolerate high voltage due to the high breakdown voltage. Figure 6 illustrates how to differentiate between diode-like TFT and a typical Schottky diode based on the rectification ratio.

Thanks to ITO transparent electrode coated atop, device functions as a dual-mode harvester module as it is exposed to any kind of light sources. Photon absorption on 3D photosensitive DGTFT increases charge concentration in active channel and leads to decreasing internal resistance of TFT.

(10) $$R_{TFT}=\frac{V_{DS}}{I_{D}}=\frac{V_{TG}}{\frac{\mu_{FE}C_{Bottom}W}{2L}{\left\{V_{TG}-V_{T_{0}}-\gamma [V_{TG}+\frac{\left[q\phi_{\lambda }(1-R)(1-e^{-\alpha t})\right]T_{s}}{C_{Top}}]\right\}}^{2}},$$

where *µ_FE_* is the field effect mobility, *W*, and *L* are width and length of TFT active channel, *C_Bottom_* is the bottom capacitance, *V_T0_* is the threshold voltage of the DGTFT under zero force applied to the PVDF, γ is the dependence factor which shows well how *V_T_* depends on *V_TG_*, $\phi_{\lambda }$ is the photon flux at a certain wavelength (λ), R is the reflectance loss in percentage, α is the absorption coefficient of the a-Si:H, *t* is the thickness of the a-Si:H, q is the elementary electron charge and the term $(1-e^{-\alpha t}$) gives the internal quantum efficiency. $T_{s}$ is the light exposure time in seconds. 

**Figure 7:**
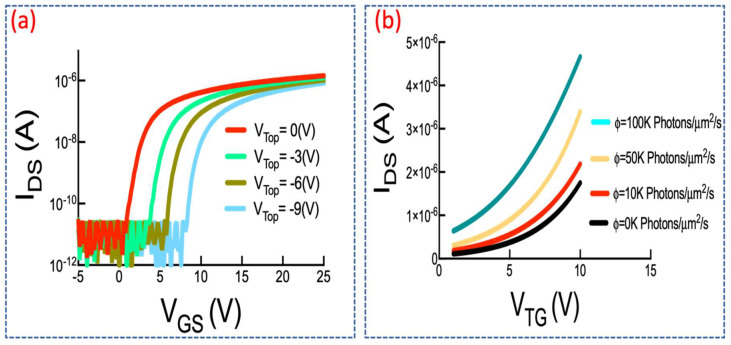
(**a**) IV curve of DGTFT under various top-gate biases; (**b**) TFT characteristics under different photon fluxes.

Characteristics of fabricated DGTFT plus impact of exposure to monochromic light have both been investigated in Figure 7a,b respectively. Figure 7b states how photosensitivity is observed as the top gate of TFT is variously biased under exposure to different light intensities and curve shifting occurs in diverse conditions (*V_TH_* shifts negatively). A detail study on impact of ambient light over such dual-mode scavenger as a single module can be found in reference [33].

#### 2.2.3. A System-Level Analysis of the Dual-Mode Harvesting Glove

**Figure 8:**
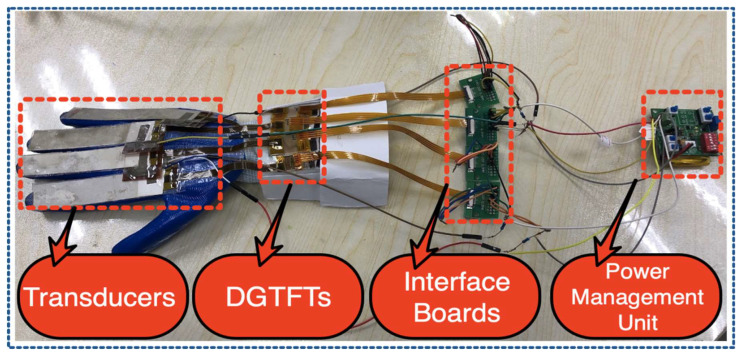
Integrated harvesting system as a Glove. Different components of each harvesting unit are shown.

The proposed harvesting system as a glove consists of four harvesting modules each containing a fabricated 3D photosensitive DGTFT on a glass integrated with a laminated 110-μm PVDF film. Four modules are firmly placed on four fingers and well-connected in parallel. Employing a double-side tape on a polysteren- fiber-glove-shaped gantlet enables us to attach all the modules tightly to form a hybrid harvesting glove (Figure 8).

Figure 8 explains well how the harvesting modules are interfaced with a particular type of silicon-based power management unit through several unique boards. Assuming of no internal loss in the whole integrated system rather than DGTFT’s internal resistance, efficiency of the dual-mode harvester glove can be approximated. Since photon absorption of each dual-mode module which is dependent on exposure time domain as well as frequency may vary sequentially with respect to the location of each unit, the internal resistance of each harvesting module shall not be predicated the same. As the efficiency of system is dependent on internal resistance of each PCGTFT, modeling of internal loss is highly preferable in this case. Therefore, the internal resistance of the hybrid glove can be given as:

(11) $$\frac{1}{R_{System}}=\frac{1}{R_{TFT(Module\#1)}}+\frac{1}{R_{TFT(Module\#2)}}+\frac{1}{R_{TFT(Module\#3)}}+\frac{1}{R_{TFT(Module\#4)}},$$

where *R*_*TFT* (*Module#*1)_, *R*_*TFT* (*Module#*2)_, *R*_*TFT * (*Module#*3)_, *R*_*TFT* (*Module#*4)_ are the internal resistance of each DGTFT in each harvesting module respectively. Extraction of system efficiency requires the equivalent resistance of the harvesting glove (Supplementary File S2). 

**Figure 9:**
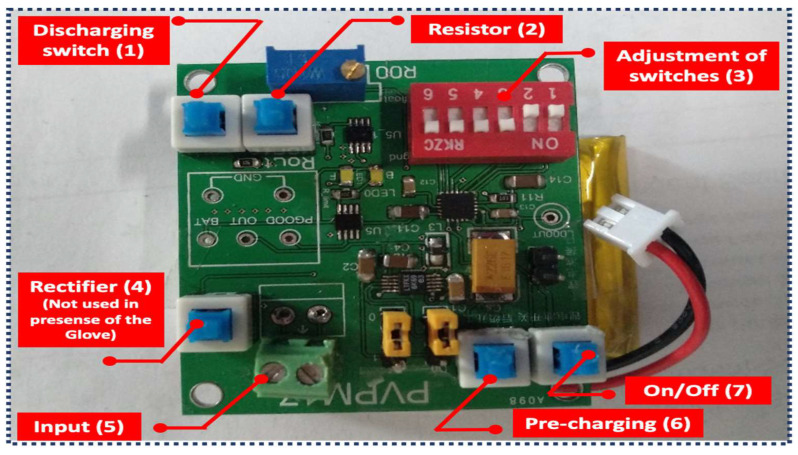
Power management box and its components as labeled with different number. Each number is described well in the manuscript.

Management of output power in wearable scavenging systems practically suffers from several issues such as interfacing, complexity of IC-circuits, flexibility, and even compatibility. Therefore, a preliminary study on design structure of such management circuit will be the next step to follow. Figure 9 describes the main components of a typical compatible management box and how it experimentally functions to manage the output power of the glove. The concept literally is based on utilizing a common super-capacitor to store energy. On one hand, the output current of hybrid system is low. On the other hand, extracted peak voltage in presence of load which varies between 4–10 V due to diverse light intensities will occur in a very short period of time. Therefore, aforementioned super-cap requires to get pre-charged through typical lithium batteries. Switch 1 (discharging switch) works well to discharge the energy harvesting (EH) capacitor. A resistor which can be tuned simply by the given screw on the blue block is connected from the positive side of the EH capacitor to the ground. To discharge the output capacitor, a resistor is also connected from the output to the ground (switch 2). Adjustment of on/off statement of all switches to change the pre-charge voltage will be implemented through switch 3. Since DGTFT acts as a rectifier in the proposed system, a designed full-bridge rectification circuit on board is off once the harvesting glove is experimentally tested (switch 4). The component labeled as 5, is the input port for energy source to power management box. Pre-charging of capacitor is practically required to reduce the time commencement of harvesting and keeps scavenging stable (switch 6). This switch also shorted with commercially bought LEDs mounted on board. Finally, to turn on/off the power management system, switch 7 must be pressed.

In simple words, the power management system functions in that any gentle flexion generates charges due to transducer compression or expansion. The obtained AC signal is rectified by DGTFT and then will be stored in a super capacitor to lighten the mounted LED on the board. The proposed silicon-based management unit yet faces with some limitations. It is experimentally understood that the management happens as the obtained voltage through utilizing 10 MΩ load, touches almost 4 V. The load resistor technically is placed in series with the four parallel harvesting modules. To testify the competency of the hybrid glove from energy stand point, an attempt has been put to light up low-power LED mounted on the management board after specific period of harvesting time. LED will be lightened as almost 3 min passed through dynamic flexion stimuli once the system works in a dark mode and approximately 1 min and 33 s in a dual-mode. The dual-mode phase here refers to a condition as the glove is exposed to ambient office light (1280 μW/cm^2^). 

**Figure 10:**
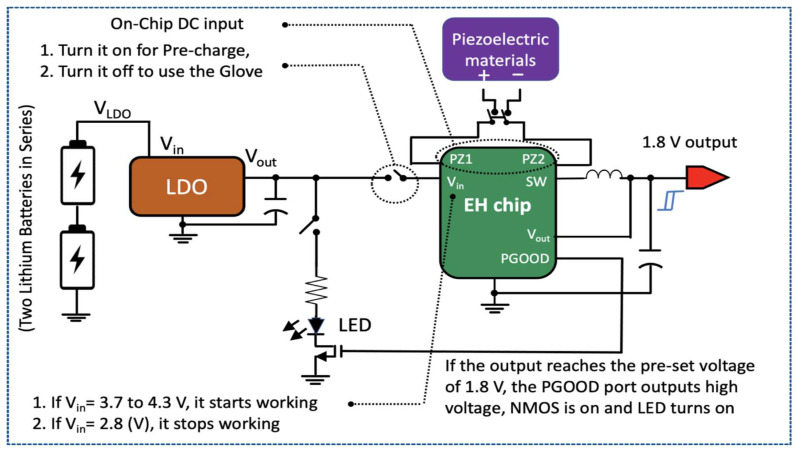
The schematic diagram of components and design layout of power management box.

The size of modern electronic devices is ever decreasing. But the battery efficiency features are changing that much which is a factor for pushing the limits on power management systems. Recently technical development in semiconductor manufacturing has led to system on-chip architecture where analog, digital and RF sub-systems are integrated into a single silicon unit. This shows that different blocks of the system have different power system requirements. A typical power management system contains several power supply circuits like: switching regulator, voltage regulator and a Low Drop out voltage (LDO). Since our power management system requires several batteries, an LDO regulator is an essential part. The output voltage (V_out_) of LDO is independent of the load impedance, the changes in input voltages (V_in_), and temperature. LDO technically can operate at very low potential difference between the input and output. In the given management circuit, two Li-ion batteries have been used which practically have a range of 4.3 V at fully charged to 2.8 V at fully discharged. Even when the batteries voltage is below 2.8 V, the LDO still maintains the desired 1.8 V at the output. Figure 10 illustrates the electrical circuit structure of power management system. A detail study of the components and functioning of this system can be found in Supplementary File S3.

**Figure 11:**
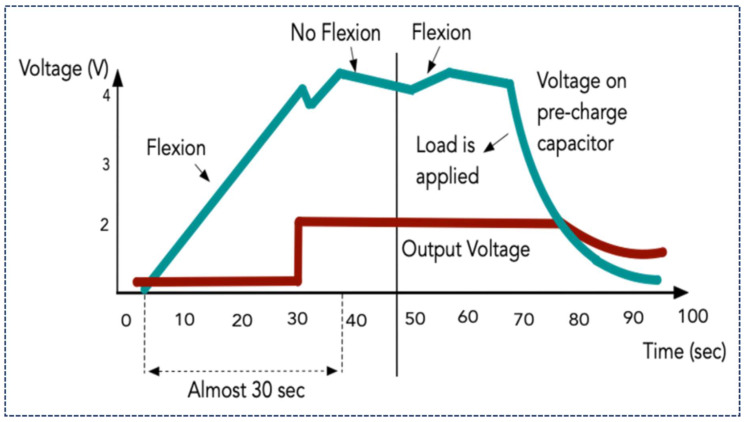
Illustration of functioning of power management unit under load.

The functionality of such a management system under a capacitive load can be explained referring to Figure 11. It clearly states the charging and discharging of the super-capacitor allocated on the management board. The red line represents the output voltage once there is no load applied while the green one shows the charging and discharging processes under an applied load. Firstly, the pre-charging occurs as the voltage touches almost 4.3 V for like 30 s through flexion. Thereafter, once the output voltage reaches the 1.8 V where the green line collides with the red one, NMOS is on and consequently the LED turns on. If the Vin ranges in 3.7 V to 4.3 V, harvesting chip starts working and as the Vin drops to 2.8 V, it stops functioning.

## 3. Results and Discussion

Commonly-used wearable single-phase harvesters practically suffer from low power conversion and efficiency. Enhancement of power in such wearables opens a new window towards hybrid harvesters by which multiple energy sources are defined for scavenging. Scavenging energy simultaneously from several distinct sources in this case would be challenging due to complexity of peripheral IC circuits and management system. Moreover, signal regulation in some particular modes of such harvesters like piezo-scavengers are essential and consequently compatibility among several modes of harvesting is considered as a key point. Proposed system unlike its counterparts overcomes complexity of IC circuits, difficulties and challenges of power management interfacing and necessity of having extra units for simultaneous harvesting.

**Figure 12:**
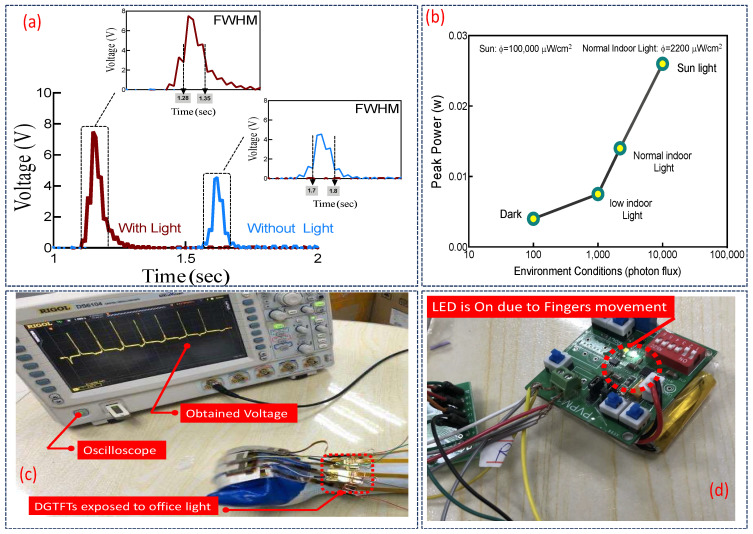
(**a**) Extracted voltage of the glove with and without office light; (**b**) Estimation of peak power of the glove under several conditions and for average daily use of 2200 cycles; (**c**) Extracted peak voltage on Oscilloscope; (**d**) Capability of the glove to lighten an LED.

A hybrid wearable glove which harvests energy from both ambient light and any dynamic stimuli along with a particular power management system are proposed. Experimentally obtained peak voltage in presence of load enhances almost 70% compared to that of single-phase glove. This enhancement further affects the peak power density and consequently efficiency of the glove. Figure 12a plots the obtained voltage in the experiment as the harvester glove is worn. On one hand, the red signal obtained where the glove is exposed to a moderate ambient office light intensity of 1280–2200 (μW/cm^2^). On the other hand, the blue line shows the glove where gentle flexions occurred in a dark place preventing any photon absorption. In both cases load resistor of 10 MΩ is utilized. Estimation of output power versus the environmental conditions in this case in presence of load is also illustrated in Figure 12b. In this scenario, internal resistance of system is assumed almost zero compared to the utilized load (ideal case). The flux is measured experimentally employing light intensity meter in each condition multiple times and the average is taken for further analysis. It is also assumed that the glove is worn daily for just average number of motions about 2500 cycles. It fairly illustrates comparatively how FWHM which stands for full width at half maximum amplitude will be reduced due to light enhancement of DGTFT since the internal resistor will be decreased drastically. As shown in Figure 12a once the glove is exposed to office light, FWHM will be 0.07 ms compared to the dark mode which is 0.1 ms (Figure 12a). This figure also illustrates that when the glove functions as a dual-mode harvester device, obtained voltage will be enhanced to almost 7.5 V while extracted voltage in dark mode touches approximately 4.2 V. This noticeable enhancement in extracted voltage across the load resistor in dual-mode glove compared to the singular-mode one leads to lessen the effective time required for system to lighten the aforesaid commercially-mounted LED through the harvested energy. Another key factor in such hybrid harvesting system is considered to be the stability and reliability of ultimate product. In this scenario, on one hand, stability of DGTFT under stress as the back bone of the glove is way more important since other components of the system are well-stable under high stress. On the other hand, as the proposed system is classified well among wearables, average daily motions of human body shall be also noted [34]. In practice, fabricated DGTFT as mounted in the system, is capable of having at least 1000 cycles of functioning which is acceptable assuming 2500 average daily grasp for an adult. Cycle here refers to gentle bending of all the four fingers at frequency of 2 to 5 [Hz]. 

Figure 12c shows the extracted voltage screening of hybrid harvester glove utilizing in-house oscilloscope (Supplementary Movie S1). Employing the power management system testifies capability of the glove to lighten an LED (Figure 12d). The effective time (to turn on the LED) lessons drastically to almost 50% in presence of light. 

Table 2 contrasts between two several types of wearable harvester with respect to utilizing different type of TFTs. It justifies employing a 3D photosensitive DGTFT over a conventional type based on the obtained output peak power either in a single-pixel or an array format. Load of 10 MΩ is utilized in all the given cases with respect to a single gentle flexion in low frequency of 2–5 [Hz]. In dual-mode harvesting scenario, harvester is exposed to ambient office light. This table also describes 20% performance improvement initially related to a comparison between an array and a single pixel device which consequently justifies formation of a dual-mode glove. Data analysis approves the improvement in output peak power almost about 47% once the conventional TFT is replaced with 3D dual-gate photosensitive TFT. The given peak power in Table 2 in each case, has been estimated through obtained peak voltage which tabulated in Table 3. Conditions to extract the peak voltage remained the same as discussed earlier for data analysis in Table 2. 

## 4. Conclusions

A system-level approach towards forming a novel hybrid harvesting glove has been discussed. This system consists of four distinct dual-mode scavengers employing a piezoelectric transducer (PVDF) incorporated with a 3-D photosensitive a-Si:H dual-gate TFT. lamination of transducer thin-film, fabrication of 3-D DGTFT, integration of each harvesting module along with formation of a hybrid harvesting glove are all well-addressed. Numerical modeling of such system to estimate the efficiency of the glove is also given. Aforementioned glove contains a particular power management system which manages the output power. Design structure and electronic components of management system along with circuits are briefly discussed. Extraction of output voltage under two various modes of the harvesting is quantified. It is shown that the proposed hybrid glove is capable of lightening a commercially-available LED. Such hybrid harvesting system is applicable in some particular human-robot interfaces, gaming, and even as the E-skin if some modifications will be made furtherly.

## Figures and Tables

**Table 1:** Summary of this work compared with the prior state-of-the-arts.

Type of Harvester	Output Type	Rectification	Max. Power Tracking Point Circuit	Application	Extra Units in Management	Interfacing between Different Modes of Harvesting	Ref.
PV	DC	NO	Required	Wearable Wrist band	NO	NO	[24]
PENG	AC	YES	Required	shoe	NO	NO	[25]
TENG	DC	NO	YES	Wearable medical devices	YES (DC-DC booster)	NO	[26]
RF	AC	YES	YES	WIFI	YES (Booster)	NO	[27]
PV-PENG	AC & DC	Needed for PENG	YES	Sensor (Working with Wind and Light)	YES	YES (Challenging and Complexed)	[28]
PV-TENG	AC & DC	Needed for TEG	YES	Enhancement of indoor WSN	NO (Each harvesting mode needs a unique management unit)	YES (Mismatch between harvesters occurs)	[29]
PENG-TENG	AC	YES	NO	Aeronautics	NO	YES (Problematic)	[30]
Solar-Dynamic Stimuli	AC	NO	NO	GLOVE	NO	NO	This Work

**Table 2:** Performance comparison between harvesters with conventional and 3D photosensitive DGTFT.

Comparison of	Single-Pixel Singular-Mode Harvester with Conventional TFT	Single-Pixel Dual-Mode Harvester with Photosensitive DGTFT	Array of Singular-Mode Harvester with Conventional TFT	Array of Dual-Mode Harvester with Photosensitive TFT (The Glove)
Output Power	2.8–3.4 µW	3.5–5 µW	5.8–8.8 µW	8.5–13 µW

**Table 3:** Peak voltage extraction between harvesters with conventional and 3D photosensitive DGTFT.

Comparison of	Single-Pixel Singular-Mode Harvester with Conventional TFT	Single-Pixel Dual-Mode Harvester with Photosensitive DGTFT	Array of Singular-Mode Harvester with Conventional TFT	Array of Dual-Mode Harvester with Photosensitive TFT (The Glove)
Peak Voltage	5.2–5.8 V	5.9–7.0 V	7.6–9.2 V	9.4–11.5 V

## Data Availability

Not applicable.

## Supplementary Materials

The following are available online at https://www.mdpi.com/article/10.3390/s21165349/s1, Image in File S1: Schematic of lamination process, Tabulation in File S1: Lamination parameters of operation, Video S1: Hybrid glove. Images in File S2: Poly(vinylidene fluoride) chemical formulation, Piezoelectricity and directions, Finite element analysis of a pixelated array of PVDF under several conditions and deformation along with charge surface accumulation, Effect of transducer thickness on surface charge density, Tabulation in File S2: Two various types of piezoelectric materials. Images in File S3: A typical 1.8 V low-voltage input piezoelectric power supply, 10-lead plastic DFN and Pin configuration of LTC 3855-1, MSE package (10-lead plastic eMSOP) and pin configuration, Block diagram of energy harvesting chip, Simplified circuit diagram of LDO, Tabulations in File S3: Pin configuration of MSE package and DFN, Pin configuration of LDO.
