# Supplementary material for: A System-Level Approach towards a Hybrid Energy Harvesting Glove
Source: Sensors (Basel). 2021 Aug 8;21(16):5349. doi: 10.3390/s21165349 (PMC8400813; doi:10.3390/s21165349)
Supplement: Supplementary file 1 [file sensors-21-05349-s001.zip › Supplementary File S1 final.pdf]

Supplementary File S1

1. Lamination of PVDF transducer:

The laminator machine as specifications were given earlier consists of a process chamber, a mechanical vacuum pump, a pressing device, a temperature control device and a process control system. Each component has its own duty such as the mechanical vacuum pump which can pump the process chamber to a certain vacuum state to provide a suitable air pressure environment for the process. Vacuum chamber itself contains two plates; each has equipped with temperature control. Pressure also can be controlled through a digital gauge. Temperature and pressure as the key factors in lamination can be modified through the system user interface. These parameters are shown in Table I are all modified and illustrated with a set of pre-defined values. Trial and error with respect to modifications of key points and factors in bonding resulted in having a minimum value for each which consequently leads to a successful lamination process. Optimization through trial and error gave a clear view about the pressure which needs to be set as 0.3 MPa. As the lamination process is done, testing of connection resistance may be considered through utilizing a multimeter while for conductive adhesive strength bending of transducer almost 90° will be taken into account. As mentioned earlier, since the polarization of piezoelectric material will be lost going above 75°C temperature of both plates is kept under this temperature.

Tabulation 1 Lamination parameters

Manual	Correspondence Unit	Condition and Set Value (CSV)
Pump Opening	Pump Control	
Wait Vacuum	Vacuum System	$CSV < 50\ mTorr$
Heating	Top and Bottom-Plates	CSV @ 70 °C for Top-Plate<br>CSV @ 75 °C for Bottom-Plate
Wait Temperature	Top and Bottom-Plates	CSV < 69 °C for Top-Plate<br>CSV < 74 °C for Bottom-Plate
Pressure	Dynamic Control	CSV @ 0.4 MPa
Wait time		CSV @ 7 Mins
Pressure	Dynamic Control	CSV @ 0 MPa
Wait Temperature	Top and Bottom-Plates	CSV < 100 °C for Top-Plate<br>CSV < 100 °C for Top-Plate
Contact	Dynamic Control	
Pump Off	Pump Control	CSV @ 1.01 KPa

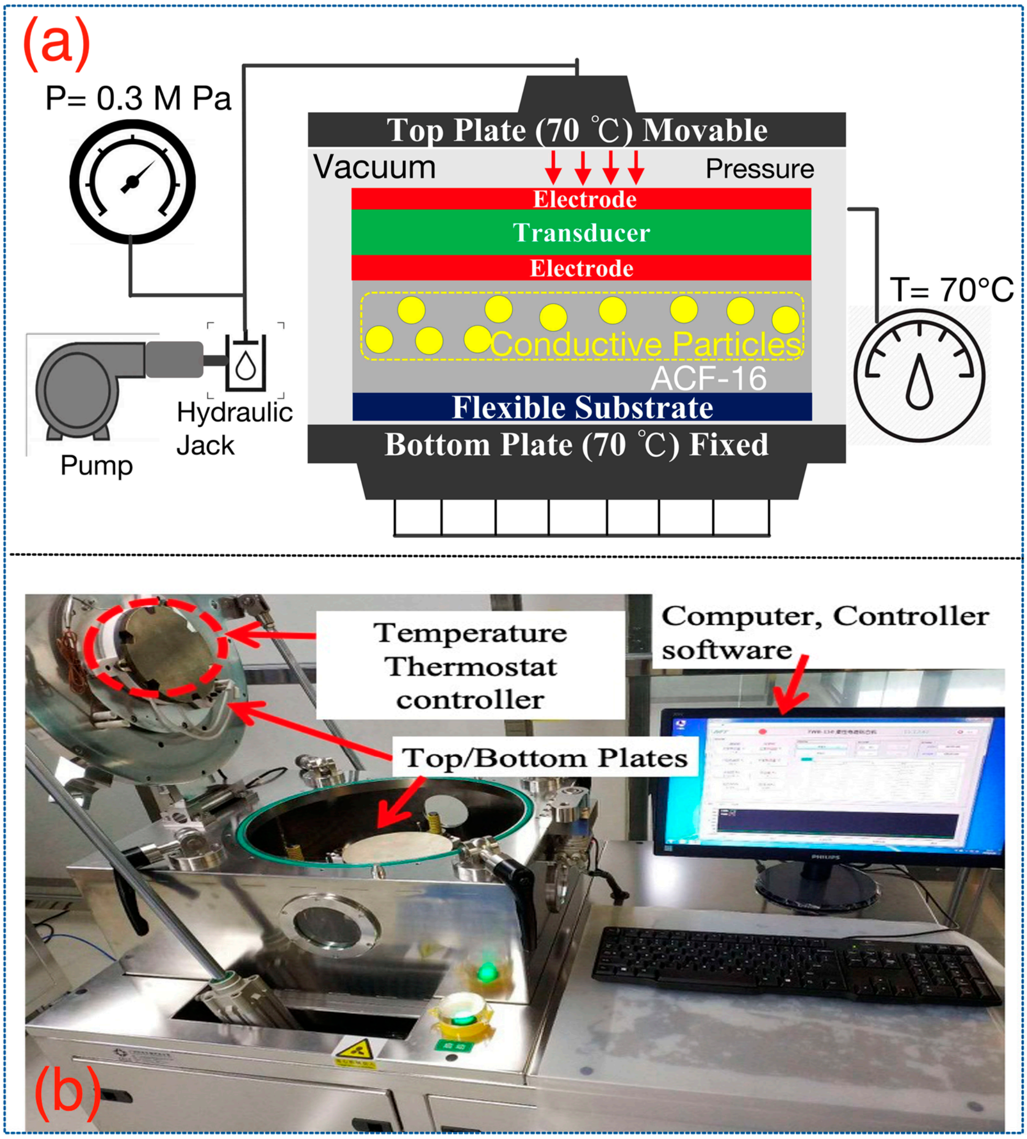

Image 1. **(a)** Schematic laminator machine and explanation of the process; **(b)** Laminator machine, TWB-150; KeFu Instrument
