# Supplementary material for: A System-Level Approach towards a Hybrid Energy Harvesting Glove
Source: Sensors (Basel). 2021 Aug 8;21(16):5349. doi: 10.3390/s21165349 (PMC8400813; doi:10.3390/s21165349)
Supplement: Supplementary file 1 [file sensors-21-05349-s001.zip › Supplementary File S2 final.pdf]

Supplementary File S2

In this supplementary file, mainly, properties of utilized PVDF as the transducer film along with system internal loss and consequently efficiency of the proposed glove will be discussed. Also, crucial factors which play an important role in the lamination process of PVDF film will be explained.

1. PVDF Characterization, properties and simulation

Scientists already reported different types of piezo-materials as tabulated and compared in Table 1. Referring to theoretical analysis, piezoelectric materials have two main coefficient factors in two different directions shown in the same table. Comparison between two widely used piezo-materials justifies utilization of PVDF as transducer in the hybrid harvesting glove. PolyVinylidene Fluoride (PVDF) is a proper candidate due to its flexibility, user-friendly, and of course reliability.

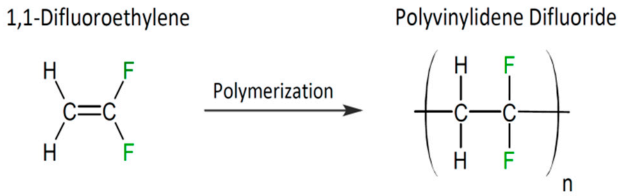

Image 1. PVDF chemical formula

Tabulation 1 Properties of two different piezoelectric materials

Property	Units	PVDF	PZT
Density	g/cm^3	1.78	7.6
Relative Permittivity	$\varepsilon/\varepsilon_0$	12	1700
Elastic Modulus	10^{10} N/m	0.3	4.9
Piezoelectric Constant	10^{-12}C/N	d_{31}=20, d_{33}=30	d_{31}=180, d_{33}=360
Coupling Constant	CV/Nm	0.11	K_{31}=0.35, K_{33}=0.69

Coefficients of piezo-materials with respect to two main directions are given in Table 1. These directions are illustrated in Figure 2.

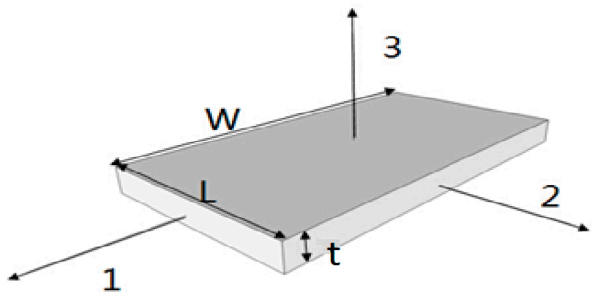

Image 2. Directions of piezoelectric coefficients

Before lamination process of PVDF transducer, simulation of an array of transducer thin film as the charge generator of the wearable harvester employing COMSOL Multyphysics will be studied in details. Since the charge distribution of PVDF film modifies as any modification occurs in applied load therefore, a complete study on this matter plays an important role since the input of dual-gate TFT as mentioned earlier is sourcing from PVDF charge generation. Simulation of such thin-film charge generator with various thicknesses would be useful for further investigations. To do so, several thicknesses of PVDF transducer are considered. The given figures below in each simulation part would be showing the boundary conditions, (how the pressure load is applied on the pixelated array of thin-film transducer), displacement and deformation of the array, and charge distribution of such pixelated array as well. To investigate how each pixel responds to the applied force, a pixelated array of PVDF was simulated to elaborate the mechanical deformation distribution and generated charge density in each pixel. In Finite Element Analysis, 100 pixels were designed in an array form, each differs from others based on the given film thickness. Dimensions of each pixel in the arrays are as 100 μm (W) ×100 μm (L) ×110 μm (T).

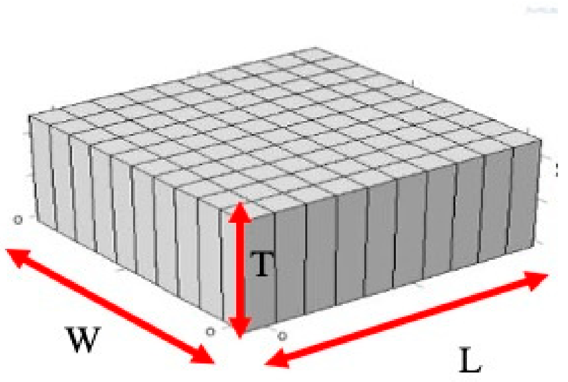

Image 3. Designed Finite Elements in a PVDF array. The size and thickness are magnified for a clear view.

To examine how each pixel behaves under different loading conditions, a particular uniform load is well applied to specific number of pixels and the rest of them were spared from the direct loading. Figures 4 (a) to (c) not only present the displacement of the array when the constant load is applied to 4 pixels uniformly from the centre respectively, but also illustrate

the charge distribution as the load pressure is kept at 10 KN/m2 and is only exerted to the top of these pixels. Load is also applied on 16, 64, and 100 pixels but not given in this supplement. PVDF thickness in this case is fixed at 110 μm. As can be seen, the displacement/deformation propagates from the centre to the edges and the central loading affects the neighbouring pixels, therefore, the similar charge distribution should be expected. The same simulation procedure with the same boundary conditions for different thicknesses as 250 μm, 500 μm, and 1000 μm will be followed. To make a study survey on the effect of thickness on charge generation of PVDF film, surface charge density generating due to applied stress on 16 pixels have been compared. Conversely to the thicker thickness as shown earlier, charge density and of course surface deformation of the PVDF film would be interestingly changed. Thinner film thickness results in growth in deformation as well as charge distribution. This phenomenon could be explained once we consider the polarization of piezo-materials and of course the coordination on which the load pressure will be exerted. Since charge distribution corresponds with the mechanical deformation upon the pressure and will ultimately make each pixel's electrical response affected and even results in power generation from those pixels where the TFTs are switched on, efficient harvesting strategy would be based on an array format.

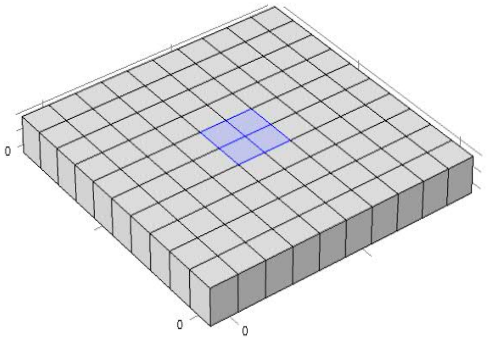

(a)

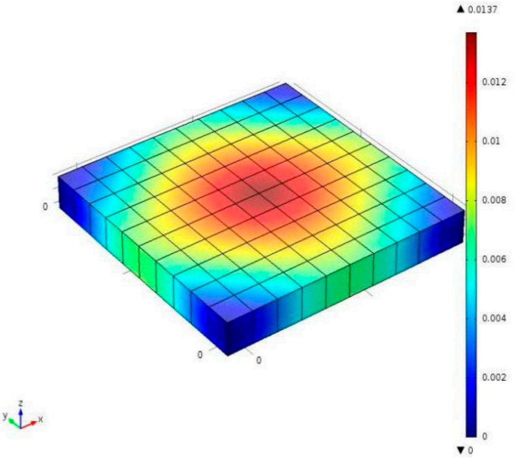

(b)

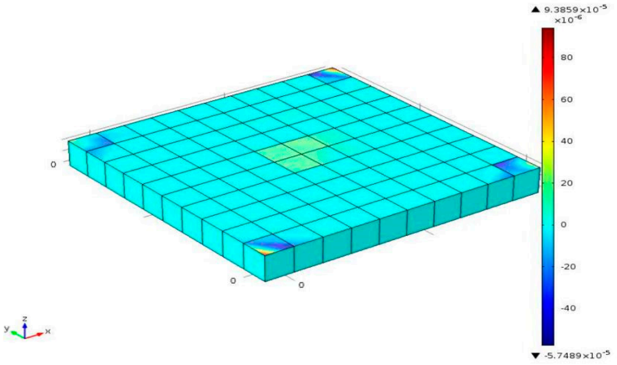

(c)

Image 4. (a) Pixelated array of PVDF ($100\mu m \times 100\mu m \times 110\mu m$) and 10 KN/m^2 is exerted on 4 pixels in

the canter uniformly; **(b)** Displacement/deformation distribution of the array with respect to (a); **(c)** Charge accumulation on the surface of the array.

Based on the given results the larger the thickness is, the less charge generates. This means that thicker film PVDF film due to less deformation would generate less charges. The deformation decreases since the amount of force is exerted on the surface is kept constant. Conversely, if the thickness of the transducer film decreases, the film deformation increases and consequently charge generation would increase drastically. To optimize the thickness and select the right thickness for the harvester in experimental analysis 110 μm PVDF film is considered.

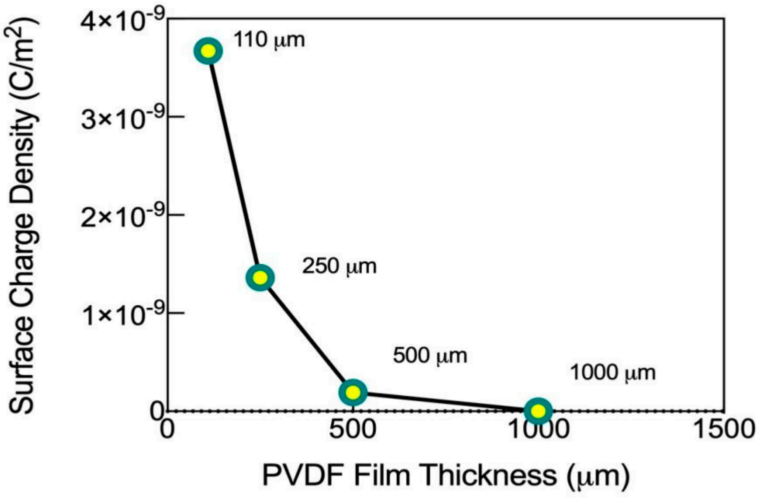

Image 5 Effect of film thickness on surface charge density as the applied force remains constant.

2. Efficiency and internal loss of the hybrid glove

Extraction of system efficiency requires the equivalent resistance of the harvesting glove. As the units are well-mounted in parallel and taking a look on Eq. 7 given in the manuscript:

$$R_{System} = \frac{[R_{TFT(Module\#2)}.R_{TFT(Module\#3)}.R_{TFT(Module\#4)}]}{R_{TFT(Module\#1)}.R_{TFT(Module\#2)}.R_{TFT(Module\#3)}.R_{TFT(Module\#4)}} + \frac{[R_{TFT(Module\#1)}.R_{TFT(Module\#3)}.R_{TFT(Module\#4)}]}{R_{TFT(Module\#1)}.R_{TFT(Module\#2)}.R_{TFT(Module\#3)}.R_{TFT(Module\#4)}} + \frac{[R_{TFT(Module\#1)}.R_{TFT(Module\#2)}.R_{TFT(Module\#4)}]}{R_{TFT(Module\#1)}.R_{TFT(Module\#2)}.R_{TFT(Module\#3)}.R_{TFT(Module\#4)}} + \frac{[R_{TFT(Module\#1)}.R_{TFT(Module\#2)}.R_{TFT(Module\#3)}]}{R_{TFT(Module\#1)}.R_{TFT(Module\#2)}.R_{TFT(Module\#3)}.R_{TFT(Module\#4)}}, \quad (1)$$

efficiency of system can be modeled referring to [33] hence,

$$\eta_{System} = \frac{1}{2}\{1+[\frac{(R_{TFT(Module\#2)}.R_{TFT(Module\#3)}.R_{TFT(Module\#4)})}{(R_{TFT(Module\#1)}.R_{TFT(Module\#2)}.R_{TFT(Module\#3)}.R_{TFT(Module\#4)}).R_L} + \frac{(R_{TFT(Module\#1)}.R_{TFT(Module\#3)}.R_{TFT(Module\#4)})}{(R_{TFT(Module\#1)}.R_{TFT(Module\#2)}.R_{TFT(Module\#3)}.R_{TFT(Module\#4)}).R_L} + \frac{(R_{TFT(Module\#1)}.R_{TFT(Module\#2)}.R_{TFT(Module\#4)})}{(R_{TFT(Module\#1)}.R_{TFT(Module\#2)}.R_{TFT(Module\#3)}.R_{TFT(Module\#4)}).R_L} + \frac{(R_{TFT(Module\#1)}.R_{TFT(Module\#2)}.R_{TFT(Module\#3)})}{(R_{TFT(Module\#1)}.R_{TFT(Module\#2)}.R_{TFT(Module\#3)}.R_{TFT(Module\#4)}).R_L}\}^{-2}, \tag{2}$$
