# Supplementary material for: A System-Level Approach towards a Hybrid Energy Harvesting Glove
Source: Sensors (Basel). 2021 Aug 8;21(16):5349. doi: 10.3390/s21165349 (PMC8400813; doi:10.3390/s21165349)
Supplement: Supplementary file 1 [file sensors-21-05349-s001.zip › Supplementary File S3 final.pdf]

Supplementary File S3

Detail study of management circuits and design

1. **Harvesting Chip**

The wearable harvesting compatible chip which was utilizing in assembling power management board is titled as LTC 3588-1. It is commercially available through Linear Technology.Co.Ltd. This chip is capable of being used in several typical harvesting applications. The typical circuit design as it was used in the proposed management board has been shown in Figure 1. Selection of the harvesting chip is based on several features and compatibility with desired application. In this case, these features can be listed as:

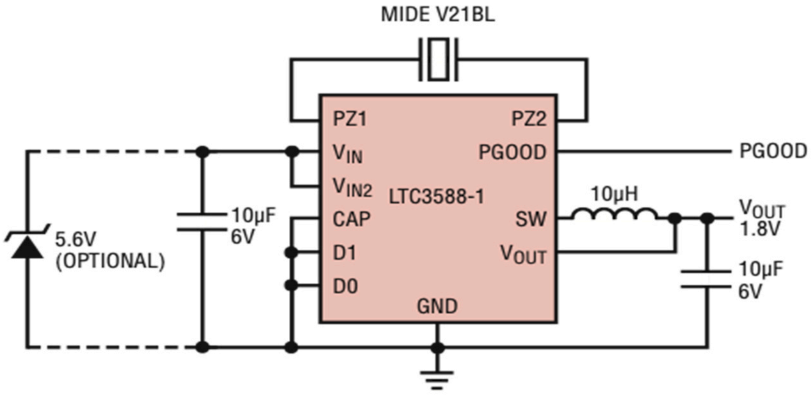

Image 1. A typical 1.8 V low-voltage input piezoelectric power supply.

a) 950 nA input quiescent current (output in regulation and no load)

b) 450 nA input quiescent current in UVLO

c) 2.7 V to 20 V operating range

d) Up to 100 mA of output current

e) Selectable output voltages of 1.8 V, 2.5 V, 3.3 V, 3.6 V

f) Wide input undervoltage lockout range (UVLO)

g) High efficiency integrated hysteretic buck DC/DC

Forming a complete energy harvesting solution for high output impedance energy sources such as piezoelectric, solar, or magnetic transducers can be done through integration of a low-loss full-wave rectifier bridge with a high efficiency buck convertor as in the given chip (LTC 3588-1). Since in the proposed harvester, 3D DGTFT acts as a half-bridge rectifier as well as a buffer, the designed full-bridge rectifier will be by-passed. To allow charges to accumulate on an input capacitor until the buck converter efficiently transfers a portion of the

stored charge to the output, an ultralow quiescent current undervoltage lockout (UVLO) was mode with a wide hysteresis window. Though we might not employ the chip for signal regulation, LTC3588-1 enters a sleep state in which both input and output quiescent currents are minimal in regulation. To maintain the regulation, the buck converter turns on and off as needed. Four output voltages, 1.8 V, 2.5 V, 3.3 V and 3.6 V, are pin selectable with up to 100 mA of continuous output current however, the output capacitor may be sized to service a higher output current burst. An input shunt which is protective will be set at 20 V which leads to greater energy storage for a given amount of input capacitance. Aforementioned chip is only available in in 10-lead MSE and 3mm × 3mm DFN. These two main parts are well addressed in Figures 2 and 3.

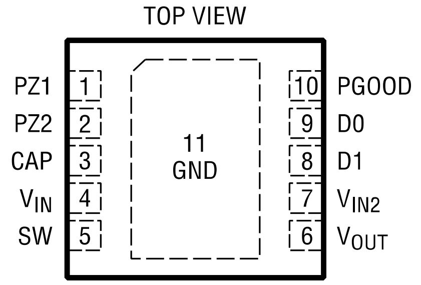

Image 2. 10-lead plastic DFN and Pin configuration of LTC 3855-1.

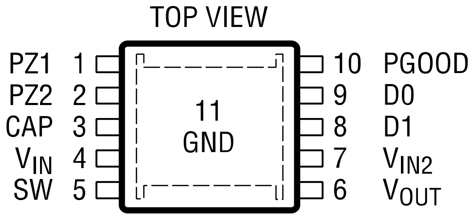

Image 3. MSE package (10-lead plastic eMSOP) and pin configuration.

Pin numbers 1 to 10 are defined based on their functionality. A glance on the pins functions makes it clear how the harvester chip works in such management system. These pins along with their configuration and numbers are all tabulated in Table 1.

Tabulation 1: Pins configuration and functionality

Title of Pin	Pin Number	Definition and Functionality
PZ1	01	Input connection for PVDF transducer (used in conjunction with PZ2)
PZ2	02	Input connection for PVDF transducer (used in conjunction with PZ2)
CAP	03	Internal rail reference to VIN to serve as gate drive for buck PMOS switch. A 1μF capacitor should be connected between CAP and VIN. This pin is not intended for use as an external system rail.
VIN	04	Rectified input voltage. A capacitor on this pin serves as an energy reservoir and input supply for buck regulator. (Since in the proposed harvester TFT acts as rectifier, rectification of such management system will not be used furtherly).
SW	05	Switch pin for the buck switching regulator. A 10μH or larger inductor should be connected from SW to VOUT.
VOUT	06	Sense pin used to monitor the output voltage and adjust it through internal feedback.
VIN2	07	Internal low voltage rail to serve as gate drive for buck NMOS. It also serves as logic high rail for output voltage select bits D0 and D1. A 4.7μF capacitor should be connected from VIN2 to ground. This pin is also designed not to use for external system rail.
D1	08	Output voltage select bit. D1 should be tied high to VIN2 or low to GND to select desired VOUT.
D0	09	Output voltage select bit. D0 should be tied high to VIN2 or low to GND to select desired VOUT.
PGOOD	10	Power good output is logic high when VOUT is above 92% of the target value. The logic high is referenced to the VOUT rail.
GND	11	It is the Ground. The exposed pad should be connected continuous ground plane on the second layer of the printed circuit board by several vias directly under the chip.

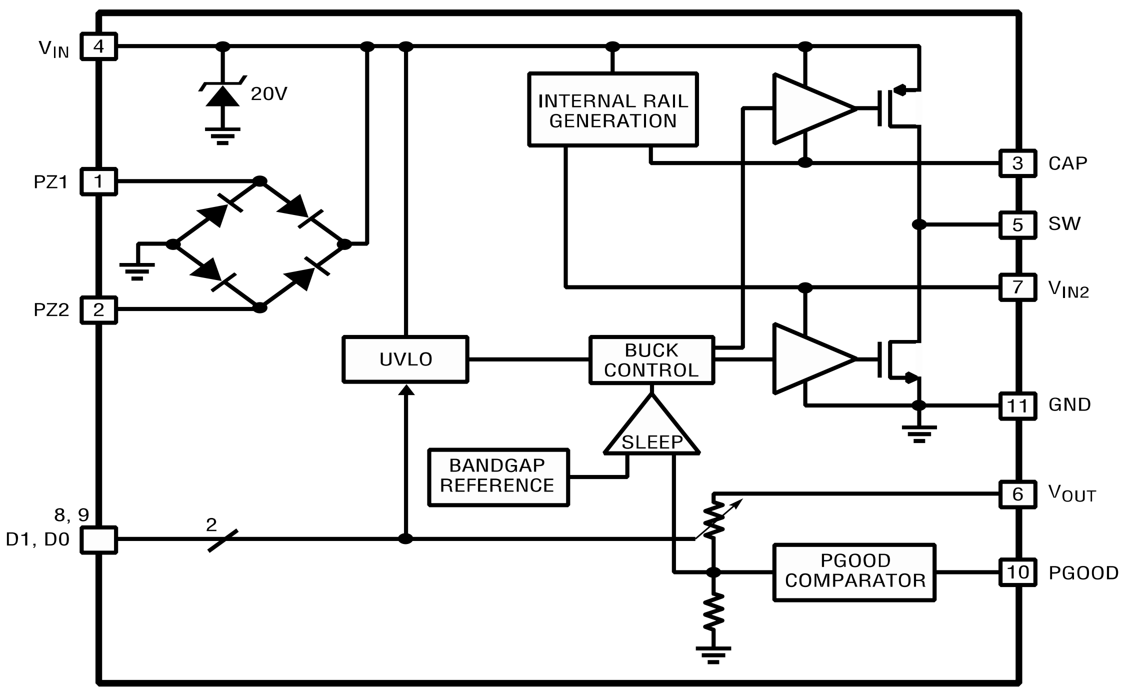

Image 4. Block diagram of energy harvesting chip.

To describe how the management circuit works we may consider the block diagram of management harvesting circuit in a typical application. The aforementioned full-bridge rectifier is accessible via the differential PZ1 and PZ2 inputs that rectifies AC inputs to DC signals (Rectification through internal full-bridge rectifier is by passed). In the case of our device, 3D DGTFT acts as rectifier and the rectified output will be stored on a capacitor at the VIN pin. This stored energy would be utilized as an energy reservoir for the buck converter. Though the internal full-bridge rectifier as mentioned earlier will not be employed in our device, the specifications of this bridge can be termed as total drop of about 400 mV with typical piezo-generated currents (~10μA) and capability of carrying up to 50 mA. It also needs to be clarified that one side of the bridge is able to function as a single-ended DC input. When the internal rectifier is in action, PZ1 and PZ2 should not be shorted. The generated charge can be transferred from the input capacitor to the output one if VIN goes beyond the UVLO rising threshold. In this case the buck converter is enabled. A wide (~1V) UVLO hysteresis window is employed with a lower threshold approximately 300 mV above the selected regulated output voltage to prevent short cycling during buck power-up. The buck convertor gets disables if the input capacitor voltage is depleted below the UVLO dropping threshold. To accumulate energy on the input capacitor an extremely low quiescent current (450 nA typical) in UVLO is considered. This practically happens once the energy is harvested from low-power sources. CAP and VIN2 contemplated as two internal rails, are generated from VIN. CAP and VIN2 are both utilized to drive the high side PMOS and low side NMOS of the buck converter respectively. Additionally, logic high for output voltage select D0 and D1 can be done by VIN2. The VIN2 rail is regulated at 4.8V above GND while the CAP rail is regulated at 4.8V below VIN. Aforementioned rails are not intended to be external. To drive the buck switches by energy reservoirs some bypass capacitors are well connected to the CAP and VIN2 pins. When VIN is below 4.8 V, VIN2 is equal to VIN and CAP is held at GND. A hysteric voltage algorithm is used in the buck regulator to have a control on the output by utilizing the internal feedback from VOUT sense pin. Using an inductor enables the buck convertor to charge an output capacitor to a value slightly higher than regulation point. This happens through

ramping the inductor current up to 260mA by employing an internal PMOS switch and then conversely ramping it down to 0mA using an internal NMOS switch and consequently energy will be delivered to the output capacitor. To check the value of the ramp, VIN, VOUT, and the inductor value are all considered. Once the UVLO threshold goes above the input voltage before the output voltage reaches regulation, the buck converter will turn off and wont function till the input voltage again rises above the UVLO rising threshold. Less than 100nA will be used to load the output voltage during this time. If the output voltage reaches the regulation, the converter enters a low quiescent current sleep state. Then it monitors the output voltage with a sleep comparator. The buck convertor provides the load current in this operation. This cycle as mentioned will be repeated if the buck regulator wakes up and as the output voltage drops below the regulation point. The buck delivers a minimum of 100mA of average load current when it is switching.

If the output reaches to the sleep threshold through monitoring by the comparator the buck converter may be in the middle of a cycle with current still flowing through the inductor. In this case, the current in the inductor would freewheel to almost zero through the NMOS diode and practically both synchronous switches would turn off. The duty of aforementioned harvesting chip (LTC3588-1) is to keep the NMOS switch on during the given operation to avoid the conduction loss that would occur in the diode once the NMOS is off. In two other cases, the NMOS will be on immediately and therefore the current will be ramped down if the PMOS is on as the sleep comparator trips. Conversely, if the NMOS is on it will be remained on till the current goes to zero. When the buck switches the quiescent current is greater than the sleep quiescent current. In most of load conditions, this still covers a small percentage of the average inductor current which leads to high efficiency. Adequate accumulated energy in the input capacitor and less energy accumulation time than the time that convertor requires for transferring the energy to the output is the thrust for the buck. Therefore, this results in low average current over a long period of time. This feature of the chip basically functions well to harvest small amount of energy. Four selectable voltages are available by tying the output select bits, D0 and D1, to GND or VIN2.

A power good comparator produces a logic high referenced to VOUT on the PGOOD pin the first time the converter reaches the sleep threshold of the programmed VOUT, signaling that the output is in regulation. Till VOUT drops to 92% of the required regulation voltage, the PGOOD pin will remain high. Moreover, if VIN goes below the UVLO falling threshold, PGOOD will also be high. It also needs to mention that PGOOD will remain high until VOUT drops to 92% of the desired regulation point. In another word, what has been discussed allows the output energy to be utilized even if the input is lost. The design of PGOOD pin is mature enough to drive a microprocessor or other chip I/O but it is not structured to be a thrust for higher current loads such as an LED.

As discussed earlier in this section, harvested energy can be stored either on the input capacitor or the output capacitor. Advantage of wide input range is related to energy storage on a capacitor which has numerical relation to the square of the capacitor voltage. All the excess energy will be stored on the input capacitor if the output voltage is regulated. The energy at high voltage can be perfectly transferred to regulated output through the buck once a typical load is presented. The load current is based on buck convertor supply whilst input energy storage utilizes the high voltage. If larger loads are required, for some duration the output capacitor can be sized. For example, when PGOOD goes high a current burst begins and will continuously deplete the output capacitor till PGOOD goes low. Till now, basic principles of such management system have been discussed. Back to a typical low-voltage piezoelectric power supply to be used with such circuit for harvesting and storage, and as in our case of study, this application is compatible with our desired target. This case as mentioned earlier, is true for output capacitors on the order of 100 μF or larger, but as the output capacitor decreases towards 10μF delays in the internal sleep comparator along with

the load current may result in the VOUT voltage slewing past the ±12 mV thresholds. This will lengthen the sleep time and increase VOUT ripple. It needs to clarify that utilizing a 10μF-capacitor or less is not demanded since VOUT ripple could be enhanced to an undesirable level. Although the designed buck is well adjusted to function along with an inductor in the range of 10 μH to 22 μH, values out of the given range may bring some benefits with respect to different applications. to be more specific, having a large inductor results in considering a beneficial high voltage applications. To have a larger inductor, the on time of PMOS switch should be enhanced. Large inductor also improves efficiency since the gate charge loss will be decreased. A 1μF capacitor should be connected between VIN and CAP and a 4.7 μF capacitor should be connected between VIN2 and GND. Not only the internal rails in buck switching are held up by the given capacitors, but also these capacitors make up the internal rail generation circuits. Therefore, in some cases as the input source is restricted by 6 V or less, the CAP and VIN2 pins are well tied to GND and the VIN respectively. This can be clearly seen in Figure 4. In order to clamp VIN, an optional 5.6 V Zener diode with leakage lower than voltage can be connected to it. No capacitor is needed on VIN2 and CAP based on the explained circuit. This story finally saves components and results in embracing a lower voltage rating for the single VIN capacitor.

2. Current Boost with Integrated LDO

An ultra-low quiescent current synchronous boost which is integrated with low-dropout regulator LDO/load switch has been commercially bought (TPS61098X) and mounted on power management board from Texas Instruments.Co.Ltd. To define the working principles of an LDO, it needs to clarify that LDO is a type of linear voltage regulator which can operate at very low potential difference between the input and the output. To solve the issues of powering some devices by either a one-cell or two-cell alkaline, NiCd or NiMH, one-cell coin cell or one-cell Li-Ion or Li-polymer battery a commercially-available TPS61098x is offered which functions well in an ultra-low-power. It integrates either a Lowdropout Linear Regulator (LDO) or a load switch with a boost converter and provides two output rails. V (MAIN) is well defined to be an always-on supply as the boost output. Unlike V(MAIN), V (SUB) is designated as the LDO switch output which has a duty to power peripheral devices. The TPS61098x has two modes controlled by MODE pin. These two modes are as: active mode and low power mode.

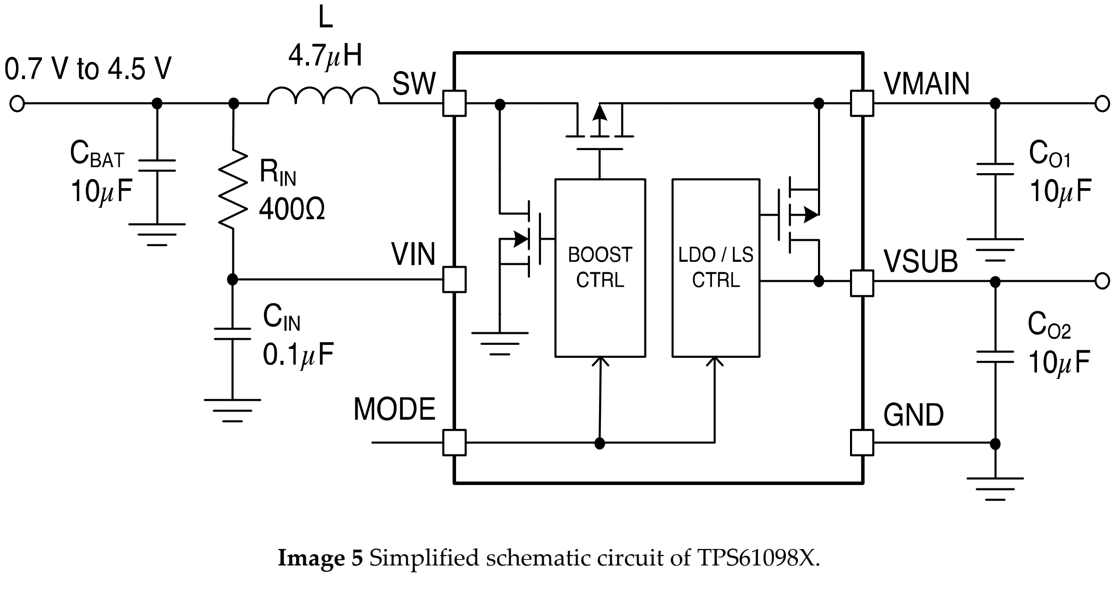

Image 5 Simplified schematic circuit of TPS61098X.

The difference between the active mode and low power mode is that in case of active one both outputs are enabled with enhanced response performance. In low-power mode, the LDO or load switch is disabled to disconnect peripherals. Having a look on data sheets of the selected booster shows that the TPS61098x consumes only 300 nA quiescent current and is able to compete with 88% efficiency at 10 μA load in low power mode. The TPS61098x supports automatic pass-through function. The booster stops switching once the input voltage gets larger than a pass-through threshold. And similarly, the booster acts in boost mode, if the threshold is higher than input voltage. It passes the input to VMAIN rail. Different output settings are offered by the TPS61098x which can be defined up to 50mA as total output current at 0.7 V input to 3.3 V output conversion. The aforementioned booster is mainly dependent upon a hysteretic controller topology utilizing synchronous rectifier to gain maximum efficiency at minimal quiescent current. The schematic of this circuit based on the given information above is given in Figure 5. The features of such circuit can be listed as:

a. Selectable output voltages Up to 4.3 V

b. Minimum 350 mA switch peak current limit

c. Integrated LDO/load switch

d. Two modes controlled by MODE pin

Active mode: Dual outputs at set values

Low- power mode: LDO/load switch off; boost keeps on

e. Automatic pass-through up to 88% efficiency at 10 μA load from 2 V to 3.3 V conversion (low-power mode)

f. Up to 93% efficiency at 5 mA ~ 100 mA load from 2 V to 3.3 V conversion

Tabulation 2: Pins configuration and functionality

Name	Pin No	I/O	Description
VMAIN	1	PWR	Boost converter output
SW	2	PWR	Connection for inductor
VIN	3	I	IC power supply input
MODE	4	I	Mode selection pin. 1: Active mode; 0: Low power mode
VSUB	5	PWR	LDO or Load switch output
GND	6	PWR	IC ground
